# Global and registry-based patterns of pancreatic cancer: GLOBOCAN 2020, CI5plus trends (2000–2017), and demographic projections to 2040

**DOI:** 10.3389/fonc.2026.1850604

**Published:** 2026-10-12

**Authors:** Xin Jin, Xiao Guan, Yongrun Mu, Min Wang, Chengfeng Wang

**Affiliations:** Department of Pancreatic and Gastric Surgery, National Cancer Center/National Clinical Research Center for Cancer/Cancer Hospital, Chinese Academy of Medical Sciences and Peking Union Medical College, Beijing, China

**Keywords:** cancer registry, CI5Plus, epidemiology, GLOBOCAN, pancreatic cancer, projection

## Abstract

**Background:**

Pancreatic cancer is a highly lethal malignancy for which mortality remains close to incidence. We combined complementary global estimates and longitudinal cancer-registry data to characterize its current burden, recent incidence trends, and demographic implications to 2040.

**Methods:**

GLOBOCAN 2020 was used for cross-sectional estimates of pancreatic cancer incidence and mortality in 185 countries and territories. Continuous annual incidence trends from 2000 to 2017 were estimated from selected non-overlapping CI5plus registry populations with complete coverage over the study period. Age-standardized rates were calculated using the Segi–Doll world standard population. Log-linear models and continuous segmented regression selected by the Bayesian information criterion were used to estimate annual percentage changes (APCs). IARC Cancer Tomorrow fixed-risk demographic projections were used to estimate incident cases through 2040; annual risk-change scenarios of −0.5% and +0.5% were examined as sensitivity analyses.

**Results:**

In 2020, an estimated 495,773 pancreatic cancer cases and 466,003 deaths occurred worldwide; the ASIR and ASMR were 4.86 and 4.53 per 100,000, respectively, and the mortality-to-incidence ratio was 0.94. Northern America and Europe had the highest regional ASIRs (8.04 and 7.77), whereas Asia contributed the largest number of cases (233,701). In pooled selected CI5plus populations, incidence increased during 2000–2006 (APC 1.43%, 95% CI 1.27–1.59) and 2006–2017 (APC 1.02%, 95% CI 0.94–1.10). The relative increase was greatest among adults younger than 50 years (APC 1.61%, 95% CI 1.37–1.85), although their absolute rate remained low. Under the fixed-risk demographic scenario, global incident cases were projected to reach 843,772 in 2040, a 70.2% increase from 2020.

**Conclusion:**

Pancreatic cancer imposes a substantial and geographically unequal burden, with mortality remaining close to incidence. Combining GLOBOCAN with CI5plus distinguishes contemporary global burden from observed registry-based trends and supports risk-based surveillance, registry strengthening, and health-system capacity planning.

## 1 Introduction

### 1.1 Disease burden and clinical characteristics

Pancreatic cancer is among the leading causes of cancer death worldwide and has one of the poorest prognoses of all solid malignancies (1, 2). GLOBOCAN 2020 estimated nearly 500,000 new cases and 466,000 deaths, illustrating the close correspondence between incidence and mortality (3). Most patients are diagnosed at advanced-stage disease, and 5-year overall survival remains poor across most global settings (4). Its poor survival largely reflects nonspecific early symptoms, late diagnosis, aggressive biology, and the limited proportion of patients eligible for curative resection (5–7). These characteristics make prevention, appropriate surveillance of high-risk individuals, and health-system preparedness particularly important.

### 1.2 Established risk factors

Established risk factors include tobacco smoking, obesity, type 2 diabetes, chronic pancreatitis, familial aggregation, and inherited cancer-predisposition syndromes (8–16). These associations provide biological and public-health context, but the present ecological analysis did not directly measure or attribute temporal changes to individual risk factors.

### 1.3 Global trends and research significance

Previous global and national studies have documented increasing pancreatic cancer incidence and marked geographic variation (17–20). However, global model-based estimates and longitudinal registry observations answer different questions and should not be treated as one continuous time series. GLOBOCAN provides standardized cross-sectional estimates that permit comparison of burden across countries, regions, development groups, and sexes, whereas CI5plus provides observed annual incidence and population denominators for high-quality registries.

The additional value of the present dual-database design is therefore methodological as well as descriptive: GLOBOCAN 2020 quantifies the contemporary global incidence and mortality burden, while CI5plus evaluates observed incidence trajectories over 2000–2017 in explicitly selected registry populations. We further link the 2020 burden to IARC demographic projections through 2040 and examine alternative annual risk-change scenarios. This separation avoids interpolating across non-continuous GLOBOCAN editions and permits transparent consideration of registry coverage and diagnostic capacity.

## 2 Materials and methods

### 2.1 Data sources

#### 2.1.1 GLOBOCAN database

GLOBOCAN 2020 estimates maintained by the International Agency for Research on Cancer (IARC) were used to describe pancreatic cancer (ICD-10 C25) incidence and mortality in 185 countries and territories (21, 22). GLOBOCAN 2020 is a cross-sectional estimation resource, not a continuous annual dataset for 2000–2020; accordingly, no interpolation or Joinpoint analysis was performed across historical GLOBOCAN editions. Country estimates are based on the best available incidence, mortality, and survival information and may involve modeling where high-quality national data are unavailable.

#### 2.1.2 CI5PLUS database

CI5plus Detailed data were used for observed annual incidence trends from 2000 to 2017 (23). Candidate registry populations were evaluated within the countries prespecified in the original study. Eligible populations were required to contain pancreatic cancer cases and corresponding population denominators for both sexes, use compatible age groups, provide continuous annual coverage throughout 2000–2017, and avoid geographical overlap with another included population. National or broad-coverage series were prioritized; where these were unavailable, non-overlapping subnational registries were pooled. The analysis included Czech Republic, Denmark, Republic of Korea, USA NPCR, Japan (Miyagi), Germany (Hamburg and Saarland), China (Jiashan, Zhongshan, and Nangang District of Harbin), India (Mumbai and Chennai), and Thailand (Chiang Mai, Khon Kaen, Songkhla, and Lampang). These registry populations were not assumed to represent their entire countries.

#### 2.1.3 Population and socioeconomic data

Development-group classifications and population projections were those implemented in the GLOBOCAN 2020 and IARC Cancer Tomorrow systems. Countries were grouped into very high, high, medium, and low Human Development Index (HDI) categories. The projection analysis used the population series embedded in the IARC Cancer Tomorrow tool to maintain consistency with the GLOBOCAN baseline.

### 2.2 Statistical analysis

#### 2.2.1 Calculation of age-standardized rate

For GLOBOCAN analyses, age-standardized incidence rates (ASIRs) and age-standardized mortality rates (ASMRs) were taken as reported by IARC. For CI5plus, age-specific rates were directly standardized to the 18-group Segi–Doll world standard population (0–4 through ≥85 years). For analyses within <50, 50–69, and ≥70 years, the standard weights were normalized within each age band. Rates are reported per 100,000 person-years.

#### 2.2.2 Time trend analysis

Continuous annual CI5plus incidence data were analyzed on the logarithmic rate scale. For the pooled selected registry population, models with zero, one, or two continuous joinpoints were compared using the Bayesian information criterion, with at least four years between candidate joinpoints. Segment-specific APCs and 95% confidence intervals (CIs) were derived from the fitted slopes. Overall age-group and registry-population trends were summarized using log-linear models over 2000–2017. Statistical significance was defined by a 95% CI excluding zero.

All trend analyses used observed CI5plus annual data; GLOBOCAN 2020 estimates were not interpolated to create artificial annual observations.

#### 2.2.3 Future burden projection and sensitivity analysis

Incident cases for 2020, 2025, 2030, 2035, and 2040 were obtained from the IARC Cancer Tomorrow projection system using GLOBOCAN 2020 age-specific incidence rates and projected population structure. The primary fixed-risk scenario holds 2020 incidence rates constant, so changes in case counts reflect population growth and ageing rather than a fitted extrapolation of the CI5plus trend. As an implementation check, the 2020 case-count anchor was reproduced from the baseline age-specific rates and population structure; this check confirms computational consistency but does not constitute external validation of the long-term projections. World, regional, and HDI-group projections were obtained from their respective Cancer Tomorrow aggregate outputs. Because projections are generated separately for each aggregation level, regional and HDI-group estimates are not necessarily additive to the independently generated World total.

Because the official fixed-risk output does not provide statistical prediction intervals, robustness was examined using two transparent sensitivity scenarios in which incidence risk decreased or increased by 0.5% annually after 2020. These bounds are scenario ranges and are not interpreted as 95% prediction intervals. The analysis therefore quantifies demographic pressure and plausible directional sensitivity without claiming validation of a long-term disease-rate forecasting model.

#### 2.2.4 Stratified analyses

Analyses were stratified by HDI group, world region, sex, and age group (<50, 50–69, and ≥70 years). R software was used for data processing, direct standardization, trend modeling, and visualization; two-sided P<0.05 was considered statistically significant.

## 3 Results

### 3.1 Global burden and geographical distribution in 2020

In 2020, there were an estimated 495,773 new cases and 466,003 deaths of pancreatic cancer worldwide (**Table 1**). The ASIR was 4.86 and the ASMR was 4.53 per 100,000, yielding a mortality-to-incidence ratio of 0.94. Men accounted for 262,865 cases and 246,840 deaths and had higher rates than women (ASIR 5.71 vs 4.06; ASMR 5.35 vs 3.77). These data emphasize that mortality remains close to incidence in both sexes.

**Table 1:** Global burden of pancreatic cancer in 2020.

Sex	Incident cases	Deaths	ASIR	ASMR	Crude incidence rate	Crude mortality rate	Cumulative incidence risk to age 74 (%)	Cumulative mortality risk to age 74 (%)	Mortality-to-incidence ratio
Both sexes	495773	466003	4.86	4.53	6.36	5.98	0.55	0.51	0.94
Male	262865	246840	5.71	5.35	6.69	6.28	0.66	0.62	0.94
Female	232908	219163	4.06	3.77	6.03	5.67	0.45	0.41	0.94

ASIR, age-standardized incidence rate; ASMR, age-standardized mortality rate.

**Figure 1** shows the country-level distribution of pancreatic cancer ASIR in 2020. The highest rates were concentrated in parts of Europe, Northern America, Japan, and Uruguay, whereas many countries in Africa and South Asia had lower estimated rates. These geographic differences should be interpreted in light of variation in population age structure, registry coverage, diagnostic access, and the modeling procedures used when observed data were sparse.

**Figure 1:**
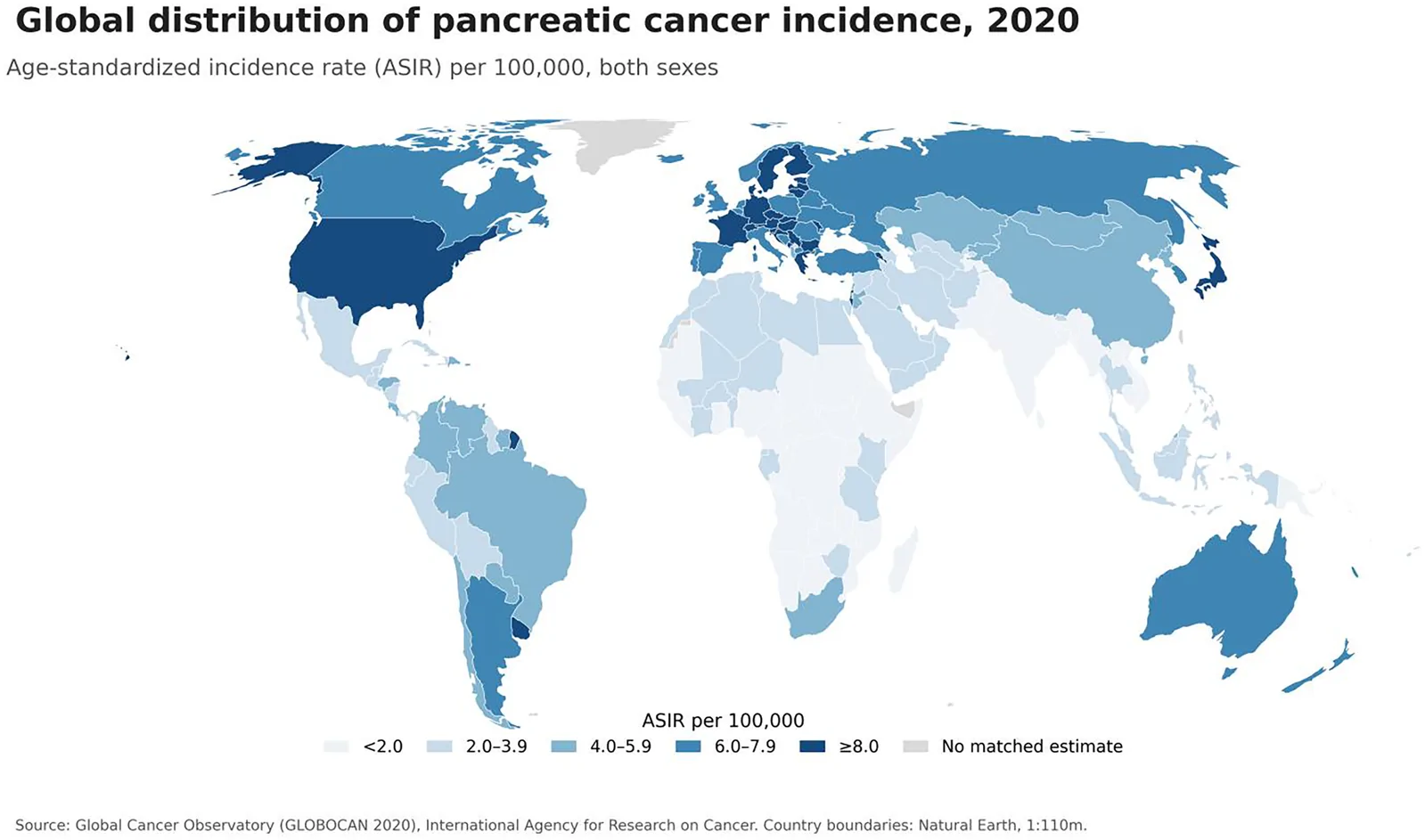
Global distribution of pancreatic cancer incidence in 2020. Country shading represents the age-standardized incidence rate (ASIR) per 100,000 for both sexes. Grey indicates no matched estimate on the 1:110m boundary map. Source: GLOBOCAN 2020; country boundaries: Natural Earth.

The map describes estimated burden rather than observed temporal change. Lower estimated rates in resource-limited settings may reflect both genuine epidemiological differences and under-ascertainment; they should not be interpreted as direct evidence of lower risk-factor exposure.

### 3.2 Incidence trends in selected CI5plus populations, 2000–2017

In the pooled selected non-overlapping CI5plus registry populations, pancreatic cancer ASIR increased from approximately 6.48 per 100,000 in 2000 to 7.84 in 2017. Segmented regression selected one joinpoint in 2006. The APC was 1.43% (95% CI 1.27–1.59) during 2000–2006 and 1.02% (95% CI 0.94–1.10) during 2006–2017 (**Table 2**). These estimates represent the included registry populations and are not global annual estimates.

**Table 2:** Temporal trend in pancreatic cancer incidence in pooled selected CI5plus registry populations, 2000–2017.

Period	APC	APC 95% CI lower	APC 95% CI upper	Model
2000–2006	1.43	1.27	1.59	BIC-selected joinpoint(s): 2006
2006–2017	1.02	0.94	1.1	BIC-selected joinpoint(s): 2006

APC, annual percentage change; CI, confidence interval. The joinpoint was selected using the Bayesian information criterion.

**Figure 2** presents the observed annual ASIRs and fitted segmented trend. The upward trajectory was sustained across the study period, although the estimated annual rate of increase was modestly lower after 2006.

**Figure 2:**
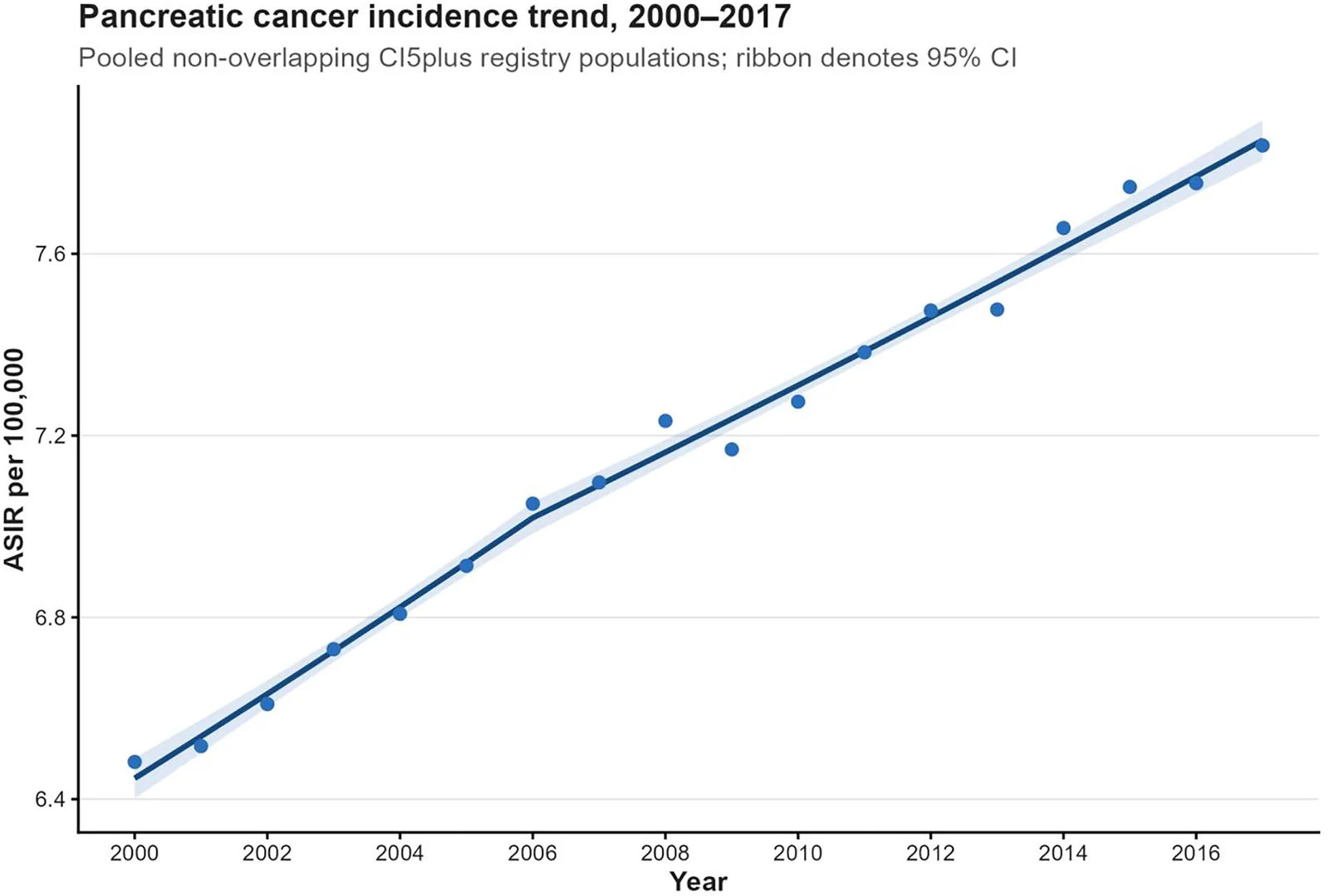
Pancreatic cancer incidence trend in pooled selected CI5plus registry populations, 2000–2017. Points are observed annual ASIRs; the line is the BIC-selected segmented fit and the ribbon is its 95% confidence band.

### 3.3 Burden and demographic projections by HDI group

In 2020, very high-HDI countries had the greatest ASIR (7.88 per 100,000) and accounted for 275,036 incident cases, followed by high-HDI countries (ASIR 4.61; 187,205 cases), low-HDI countries (ASIR 1.82; 8,586 cases), and medium-HDI countries (ASIR 1.18; 24,705 cases) (**Table 3**). Mortality showed a similar gradient, with 251,333 deaths in the very high-HDI group and 182,247 in the high-HDI group. The classification-specific estimates depend on the countries assigned to each GLOBOCAN HDI aggregate.

**Table 3:** Pancreatic cancer burden in 2020 and projected incident cases in 2040 by HDI group.

HDI group	Incident cases	Deaths	ASIR	ASMR	Crude incidence rate	Crude mortality rate	Projected cases 2040	Lower sensitivity scenario 2040	Upper sensitivity scenario 2040	Increase from 2020 (%)
Very high HDI	275036	251333	7.88	6.93	17.58	16.07	382928	346401	423095	39.2
High HDI	187205	182247	4.61	4.48	6.43	6.26	336486	304389	371782	79.7
Medium HDI	24705	23846	1.18	1.14	1.06	1.02	43463	39317	48022	75.9
Low HDI	8586	8355	1.82	1.79	0.87	0.84	17647	15964	19498	105.5

HDI, Human Development Index; ASIR, age-standardized incidence rate; ASMR, age-standardized mortality rate. Projection bounds are ±0.5% annual risk-change sensitivity scenarios, not 95% prediction intervals. World, regional, and HDI-group projections are generated separately and are not necessarily additive across aggregation levels.

Under the fixed-risk demographic scenario, incident cases increased in every HDI group between 2020 and 2040. The projected increases were 39.2% in very high-HDI, 79.7% in high-HDI, 75.9% in medium-HDI, and 105.5% in low-HDI groups. These changes primarily reflect projected population growth and ageing and do not demonstrate that underlying incidence rates will increase.

**Figure 3** displays the fixed-risk projection together with annual risk-change sensitivity scenarios. The scenario range widens with projection horizon, illustrating the dependence of long-term case counts on assumptions about future disease risk.

**Figure 3:**
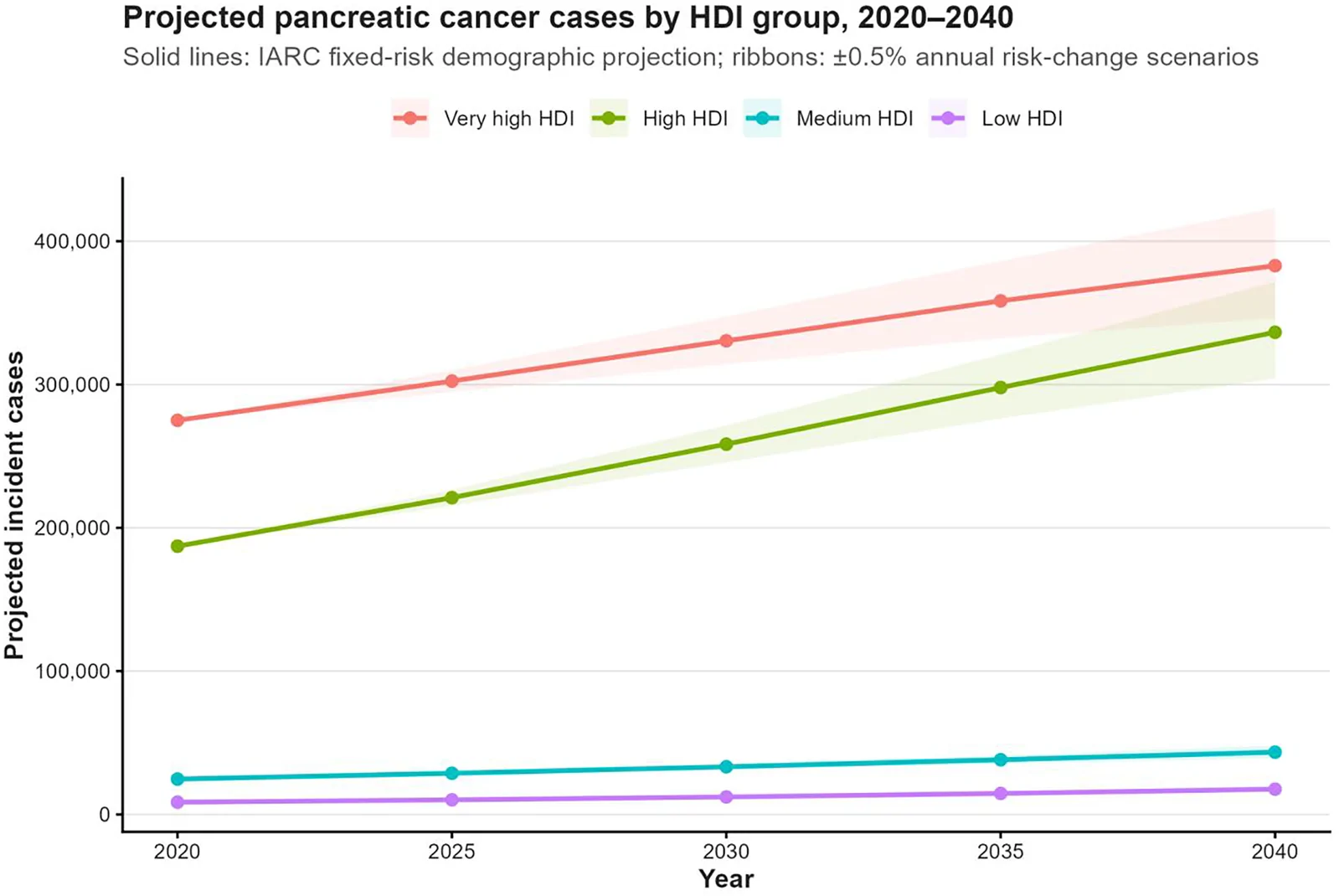
Projected pancreatic cancer incident cases by HDI group, 2020–2040. Solid lines show the IARC fixed-risk demographic projection; ribbons show sensitivity scenarios assuming annual risk changes of −0.5% and +0.5% and are not 95% prediction intervals.

### 3.4 Regional incidence and mortality in 2020

Regional burden varied substantially in 2020 (**Table 4**). Northern America had the highest ASIR (8.04 per 100,000), followed by Europe (7.77) and Oceania (6.63). Asia contributed the largest number of incident cases (233,701) and deaths (224,034), despite a lower ASIR of 3.99. Europe recorded 140,116 cases and 132,134 deaths. Mortality-to-incidence ratios ranged from 0.81 in Oceania to 0.97 in Africa, although cross-region comparisons may also reflect differences in diagnosis, certification of cause of death, and estimation quality.

**Table 4:** Pancreatic cancer incidence and mortality by world region, 2020.

Region	Incident cases	Deaths	ASIR	ASMR	Crude incidence rate	Crude mortality rate	Cumulative incidence risk to age 74 (%)	Cumulative mortality risk to age 74 (%)	Mortality-to-incidence ratio
Northern America	62643	53277	8.04	6.53	16.98	14.44	0.95	0.77	0.85
Europe	140116	132134	7.77	7.16	18.71	17.65	0.91	0.84	0.94
Oceania	4891	3979	6.63	5.2	11.46	9.32	0.76	0.58	0.81
Latin America and the Caribbean	37352	36030	4.49	4.31	5.71	5.51	0.51	0.49	0.96
Asia	233701	224034	3.99	3.82	5.04	4.83	0.45	0.42	0.96
Africa	17070	16549	2.31	2.25	1.27	1.23	0.27	0.26	0.97

ASIR, age-standardized incidence rate; ASMR, age-standardized mortality rate.

The close correspondence of ASMR and ASIR across regions reinforces the clinical lethality of pancreatic cancer and the limited effect of current early-detection and treatment capacity at population level. Mortality estimates are presented as a cross-sectional complement to incidence; annual mortality trends were not modeled because CI5plus contains incidence rather than mortality data.

**Figure 4** compares regional ASIR and ASMR. Northern America and Europe had the highest incidence and mortality rates, while Africa had the lowest estimated rates. The consistently small gap between incidence and mortality contrasts with cancers for which long-term survival is more common.

**Figure 4:**
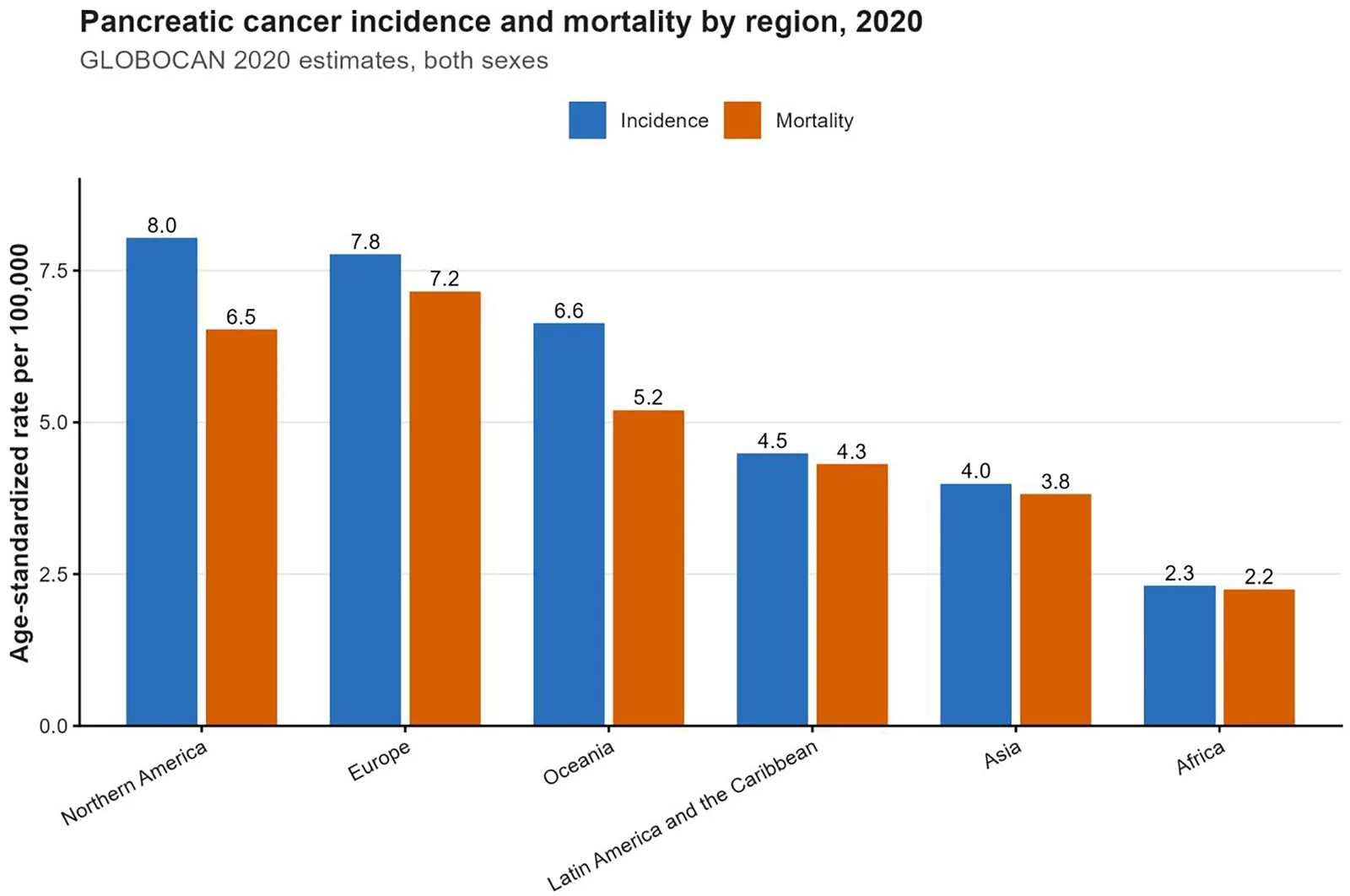
Pancreatic cancer incidence and mortality by world region, 2020. Rates are age-standardized per 100,000 for both sexes.

### 3.5 Sex differences in pancreatic cancer incidence

In 2020, male ASIR exceeded female ASIR in every region (**Table 5**). The male-to-female ratio was highest in Europe (1.48) and Asia (1.45), followed by Africa (1.36), Northern America (1.35), Latin America and the Caribbean (1.25), and Oceania (1.23). These cross-sectional comparisons do not establish temporal widening or narrowing of sex disparities.

**Table 5:** Sex differences in pancreatic cancer incidence by world region, 2020.

Region	Female	Male	Male-to-female ASIR ratio
Europe	6.37	9.43	1.48
Northern America	6.91	9.3	1.35
Oceania	5.96	7.34	1.23
Latin America and the Caribbean	4.02	5.04	1.25
Asia	3.26	4.74	1.45
Africa	1.98	2.7	1.36

Data source: GLOBOCAN 2020. ASIR, age-standardized incidence rate. ASIRs are per 100,000 population. The male-to-female ratio was calculated by dividing the male ASIR by the female ASIR.

**Figure 5** displays regional ASIRs by sex. The absolute difference was greatest in Europe and Northern America, which also had the highest overall regional incidence rates.

**Figure 5:**
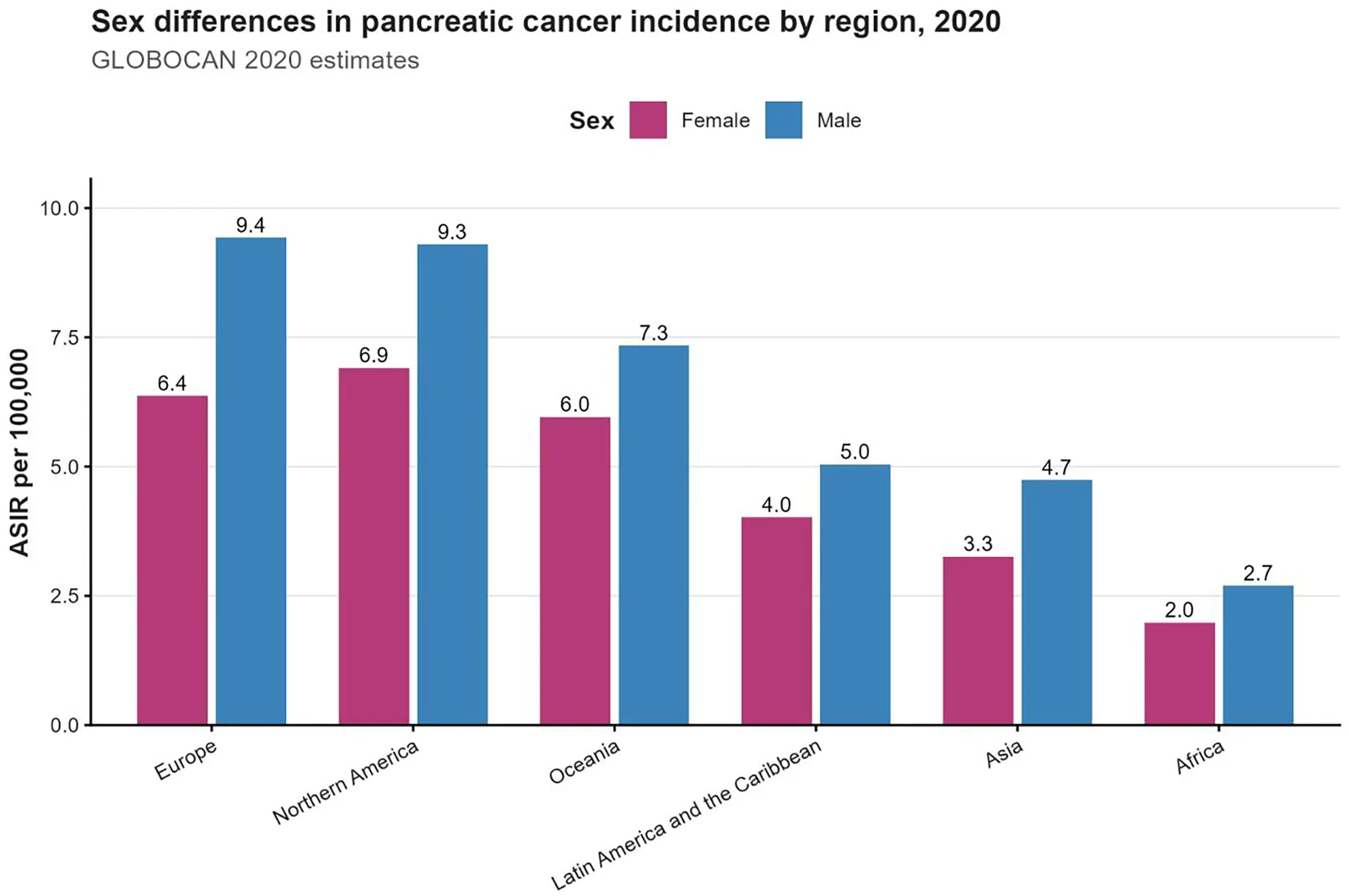
Sex differences in pancreatic cancer incidence by world region, 2020.

### 3.6 Age-group incidence trends in selected CI5plus populations

Across the selected CI5plus populations, the standardized rate within each age band remained highest among adults aged ≥70 years, increasing from 62.881 per 100,000 in 2000 to 75.242 in 2017 (APC 0.971%, 95% CI 0.845–1.097). The corresponding rate increased from 21.249 to 25.605 among those aged 50–69 years (APC 1.192%, 95% CI 1.112–1.272) and from 0.709 to 0.915 among those younger than 50 years (APC 1.610%, 95% CI 1.370–1.851) (**Table 6**). Thus, the youngest group had the largest relative annual increase but the lowest absolute burden.

**Table 6:** Pancreatic cancer incidence trends by age group in pooled selected CI5plus registry populations, 2000–2017.

Age group	ASIR 2000	ASIR 2017	APC	APC 95% CI lower	APC 95% CI upper	P value	Significant
50–69 years	21.249	25.605	1.192	1.112	1.272	<0.001	Yes
<50 years	0.709	0.915	1.61	1.37	1.851	<0.001	Yes
≥70 years	62.881	75.242	0.971	0.845	1.097	<0.001	Yes

Rates were standardized within each age band using normalized Segi–Doll weights. APC, annual percentage change; CI, confidence interval.

**Figure 6** shows a persistent age gradient throughout 2000–2017. All three groups increased, but the scale of the figure also demonstrates that a faster relative trend among younger adults does not imply a greater absolute rate than in older adults.

**Figure 6:**
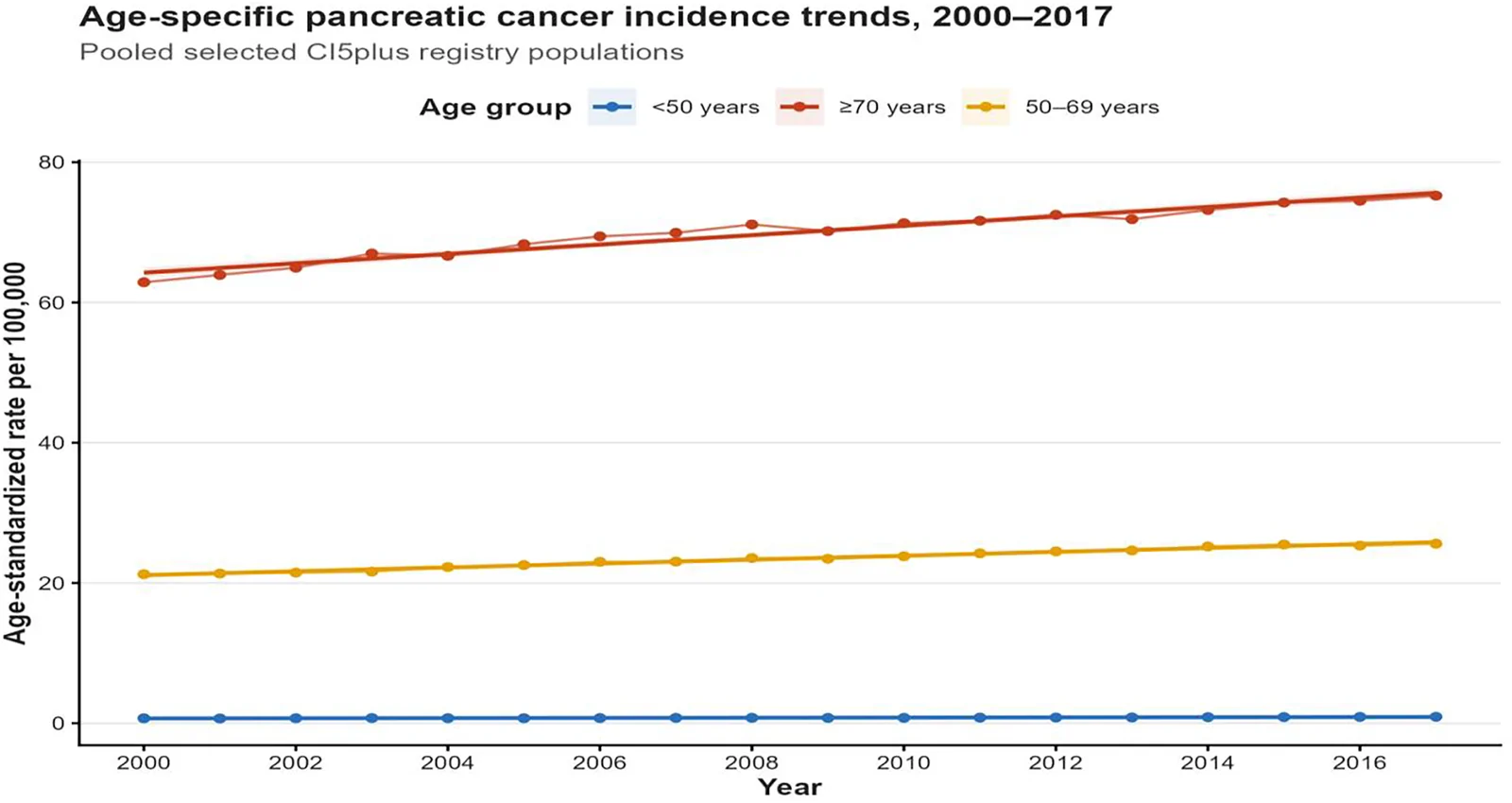
Pancreatic cancer incidence trends by age group in pooled selected CI5plus registry populations, 2000–2017.

### 3.7 Registry-population incidence trends based on CI5plus

Nine analysis populations met the prespecified continuity and non-overlap criteria (**Table 7**). In 2017, ASIR was highest in Japan (Miyagi; 10.533 per 100,000), followed by Czech Republic (9.658), Denmark (8.340), Germany (Hamburg and Saarland; 8.209), and USA NPCR (8.115). Significant increasing trends were observed in seven populations, with the largest APCs in India (Mumbai and Chennai; 2.958%, 95% CI 2.259–3.662) and Thailand (four registries; 2.934%, 95% CI 2.016–3.859). The trend was not statistically significant in Denmark or the three pooled Chinese registries. These results describe the included registry catchments rather than complete national populations except where national or broad-coverage series were available.

**Table 7:** Pancreatic cancer incidence trends in selected CI5plus populations, 2000–2017.

Analysis population	ASIR 2000	ASIR 2017	APC	APC 95% CI lower	APC 95% CI upper	P value	Significant
Japan (Miyagi)	8.303	10.533	1.402	0.814	1.993	<0.001	Yes
Czech Republic	8.845	9.658	0.533	0.233	0.833	0.002	Yes
Denmark	7.189	8.34	0.311	-0.213	0.838	0.227	No
Germany (2 registries)	7.531	8.209	0.971	0.287	1.66	0.008	Yes
USA (NPCR)	6.653	8.115	1.215	1.14	1.29	<0.001	Yes
Republic of Korea	5.547	7.245	1.52	1.361	1.68	<0.001	Yes
China (3 registries)	4.255	5.093	0.024	-1.012	1.07	0.962	No
Thailand (4 registries)	1.217	2.301	2.934	2.016	3.859	<0.001	Yes
India (2 registries)	1.36	2.177	2.958	2.259	3.662	<0.001	Yes

Data source: CI5plus. ASIR, age-standardized incidence rate; APC, annual percentage change; CI, confidence interval. Czech Republic, Denmark, Republic of Korea, and USA NPCR represent national or broad population coverage; other estimates represent the named selected registries and must not be interpreted as complete national estimates. APC was estimated from log-linear regression over 2000–2017.

### 3.8 Projected pancreatic cancer case burden, 2020–2040

Under the IARC fixed-risk demographic scenario, global incident cases were projected to increase from 495,773 in 2020 to 572,329 in 2025, 657,584 in 2030, and 843,772 in 2040, representing a 70.2% increase (**Table 8**). Asia had the largest projected absolute burden in 2040 (424,138 cases), while projected relative increases were greatest in Africa (100.1%), Latin America and the Caribbean (81.6%), and Asia (81.5%). Europe had the smallest projected relative increase (27.4%). These estimates represent demographic projections rather than forecasts of changing incidence rates.

**Table 8:** Projected pancreatic cancer incident cases globally and by world region, 2020–2040.

Area	Cases 2020	Cases 2025	Cases 2030	Cases 2040	Increase 2020–2040 (%)	Lower sensitivity scenario 2040	Upper sensitivity scenario 2040
Worldwide	495773	572329	657584	843772	70.2	763285	932280
Asia	233701	275390	321786	424138	81.5	383680	468628
Europe	140116	150137	160250	178438	27.4	161417	197155
Northern America	62643	69699	76741	89124	42.3	80623	98473
Latin America and the Caribbean	37352	43866	51207	67836	81.6	61365	74952
Africa	17070	20228	24079	34165	100.1	30906	37749
Oceania	4891	5610	6393	7933	62.2	7176	8765

The primary estimates use fixed 2020 incidence rates. Lower and upper values are −0.5% and +0.5% annual risk-change sensitivity scenarios and are not 95% prediction intervals. World, regional, and HDI-group projections are generated separately and are not necessarily additive across aggregation levels.

## 4 Discussion

This study separates contemporary global burden from observed longitudinal trends by combining two complementary IARC resources. GLOBOCAN 2020 provides comparable cross-sectional incidence and mortality estimates across 185 countries and territories, whereas CI5plus provides annual observed incidence and population denominators for selected high-quality registries. This design avoids treating non-continuous GLOBOCAN editions as a 21-year annual series and adds value beyond a single global burden analysis by linking geographic burden, registry-based trends, age and sex patterns, mortality, and demographic projections within explicitly stated data boundaries.

The geographic pattern was broadly consistent with previous global studies, with higher estimated ASIRs in Northern America, Europe, and Oceania and the largest number of cases in Asia (3, 24, 25). Possible explanations include differences in population ageing, historical exposure patterns, diagnostic practice, and registry completeness. However, smoking, obesity, diabetes, diet, and other exposures were not measured in this study; consequently, they should be regarded as plausible contextual factors rather than demonstrated drivers of the observed differences. Comparisons involving lower-resource settings require particular caution because limited diagnostic imaging, pathology access, death certification, and cancer-registration coverage may reduce case ascertainment or increase reliance on modeled estimates (26–31).

Men had higher estimated incidence than women in all regions in 2020. Differences in tobacco exposure, occupational risks, metabolic factors, health-care use, and biological susceptibility may contribute (32–40), but the present cross-sectional analysis cannot attribute the sex disparity to any specific factor or determine whether the gap is widening. The regional pattern supports sex-disaggregated surveillance and prevention while avoiding causal interpretation of ecological contrasts.

The largest relative incidence increase in the selected CI5plus populations occurred among adults younger than 50 years. Rising obesity, earlier-onset type 2 diabetes, dietary change, and other early-life exposures have been proposed as possible contributors (41–46), but none was directly analyzed here. Because the absolute incidence below age 50 remained low, these results do not support population-wide pancreatic cancer screening. More appropriate implications include strengthening investigation of persistent symptoms, improving risk assessment, and considering surveillance within established high-risk groups such as individuals with hereditary syndromes, strong family history, or selected pancreatic conditions (47, 48).

The fixed-risk projection indicates that population growth and ageing alone could increase global incident cases by approximately 70% by 2040. The scenario analysis shows that even small sustained changes in incidence risk materially affect long-term case counts. These projections were anchored to the 2020 GLOBOCAN burden but were not externally validated against future observations; they should therefore be used for capacity planning rather than interpreted as precise forecasts. Regions with rapid demographic expansion may need greater diagnostic, surgical, oncological, palliative-care, and registry capacity, while very high-HDI settings will continue to face substantial absolute burden.

The near equivalence of incidence and mortality is a central finding rather than a secondary descriptor. It reflects the poor population-level prognosis of pancreatic cancer and supports planning across the full care pathway, including timely diagnostic work-up, referral to high-volume multidisciplinary services, access to systemic therapy, supportive and palliative care, and accurate cause-of-death reporting. Surveillance priorities include improving population-based registration in under-covered settings, monitoring age- and sex-specific incidence, and linking cancer registries with mortality and clinical data where feasible. Screening should remain targeted to evidence-based high-risk groups rather than the general population.

Several limitations warrant emphasis. First, GLOBOCAN estimates depend on heterogeneous country data sources and modeling strategies; differences in registration completeness, diagnostic capacity, and mortality certification can affect both geographic comparisons and estimated mortality-to-incidence ratios. Second, CI5plus contains selected registry populations, many of which are subnational, and the pooled trend is not globally representative. Registry eligibility required continuous data through 2017 and may favor better-resourced settings. Third, risk factors were not directly measured, so etiologic explanations are contextual and cannot be inferred from these ecological results. Fourth, the fixed-risk projection assumes unchanged 2020 incidence rates; its sensitivity bounds are scenarios rather than statistical prediction intervals, and no external forecast validation was possible. Aggregate projections were generated separately and are not necessarily additive across World, regional, and HDI-group levels. Finally, annual mortality trends were not analyzed because the selected longitudinal source contained incidence only. These constraints narrow the interpretation but improve transparency and reproducibility.

## 5 Conclusion

This dual-database analysis shows that pancreatic cancer produced 495,773 incident cases and 466,003 deaths worldwide in 2020, with substantial regional, sex, and age disparities. Incidence increased during 2000–2017 across the pooled selected CI5plus populations, with the greatest relative increase among adults younger than 50 years but the highest absolute rates among older adults. Under a fixed-risk demographic scenario, global incident cases may reach approximately 843,772 by 2040. Strengthened cancer registration, risk-based surveillance, timely diagnosis, and coordinated treatment and palliative-care capacity are priorities, particularly in rapidly growing and under-resourced health systems.

## Data Availability

The original contributions presented in the study are included in the article/supplementary material. Further inquiries can be directed to the corresponding authors.
